# Gene–environment interactions involving functional variants: Results from the Breast Cancer Association Consortium

**DOI:** 10.1002/ijc.30859

**Published:** 2017-08-11

**Authors:** Myrto Barrdahl, Anja Rudolph, John L. Hopper, Melissa C. Southey, Annegien Broeks, Peter A. Fasching, Matthias W. Beckmann, Manuela Gago‐Dominguez, J. Esteban Castelao, Pascal Guénel, Thérèse Truong, Stig E. Bojesen, Susan M. Gapstur, Mia M. Gaudet, Hermann Brenner, Volker Arndt, Hiltrud Brauch, Ute Hamann, Arto Mannermaa, Diether Lambrechts, Lynn Jongen, Dieter Flesch‐Janys, Kathrin Thoene, Fergus J. Couch, Graham G. Giles, Jacques Simard, Mark S. Goldberg, Jonine Figueroa, Kyriaki Michailidou, Manjeet K. Bolla, Joe Dennis, Qin Wang, Ursula Eilber, Sabine Behrens, Kamila Czene, Per Hall, Angela Cox, Simon Cross, Anthony Swerdlow, Minouk J. Schoemaker, Alison M. Dunning, Rudolf Kaaks, Paul D.P. Pharoah, Marjanka Schmidt, Montserrat Garcia‐Closas, Douglas F. Easton, Roger L. Milne, Jenny Chang‐Claude

**Affiliations:** ^1^ Division of Cancer Epidemiology German Cancer Research Center (DKFZ) Heidelberg Germany; ^2^ Centre for Epidemiology and Biostatistics Melbourne School of Population and Global Health, The University of Melbourne Melbourne VIC Australia; ^3^ Department of Pathology The University of Melbourne Melbourne VIC Australia; ^4^ Division of Molecular Pathology Netherlands Cancer Institute–Antoni van Leeuwenhoek Hospital Amsterdam The Netherlands; ^5^ Department of Gynaecology and Obstetrics University Hospital Erlangen, Friedrich‐Alexander University Erlangen‐Nuremberg, Comprehensive Cancer Center Erlangen‐EMN Erlangen Germany; ^6^ David Geffen School of Medicine Department of Medicine Division of Hematology and Oncology, University of California at Los Angeles Los Angeles CA; ^7^ Genomic Medicine Group Galician Foundation of Genomic Medicine, Instituto de Investigación Sanitaria de Santiago de Compostela (IDIS), Complejo Hospitalario Universitario de Santiago, Servicio Galego de Saúde, SERGAS Santiago De Compostela Spain; ^8^ Moores Cancer Center University of California San Diego La Jolla CA; ^9^ Oncology and Genetics Unit Instituto de Investigación Sanitaria Galicia Sur (IISGS), Xerencia de Xestion Integrada de Vigo‐SERGAS Vigo Spain; ^10^ CESP–Cancer and Environment team, INSERM U1018, Université Paris‐Sud, Université Paris‐Saclay Villejuif France; ^11^ Copenhagen General Population Study, Herlev and Gentofte Hospital, Copenhagen University Hospital Herlev Denmark; ^12^ Department of Clinical Biochemistry Herlev and Gentofte Hospital, Copenhagen University Hospital Herlev Denmark; ^13^ Faculty of Health and Medical Sciences University of Copenhagen Copenhagen Denmark; ^14^ Epidemiology Research Program, American Cancer Society Atlanta GA; ^15^ Division of Clinical Epidemiology and Aging Research German Cancer Research Center (DKFZ) Heidelberg Germany; ^16^ Division of Preventive Oncology German Cancer Research Center (DKFZ) and National Center for Tumor Diseases (NCT) Heidelberg Germany; ^17^ German Cancer Consortium (DKTK) Heidelberg Germany; ^18^ Dr. Margarete Fischer‐Bosch‐Institute of Clinical Pharmacology Stuttgart Germany; ^19^ University of Tübingen Tübingen Germany; ^20^ Molecular Epidemiology Group, German Cancer Research Center (DKFZ) Heidelberg Germany; ^21^ Translational Cancer Research Area, University of Eastern Finland Kuopio Finland; ^22^ Pathology and Forensic Medicine Institute of Clinical Medicine, University of Eastern Finland Kuopio Finland; ^23^ Imaging Center, Department of Clinical Pathology, Kuopio University Hospital Kuopio Finland; ^24^ Vesalius Research Center, VIB Leuven Belgium; ^25^ Laboratory for Translational Genetics Department of Human Genetics, University of Leuven Leuven Belgium; ^26^ Leuven Multidisciplinary Breast Center, Department of Oncology, KU Leuven and Leuven Cancer Institute, University Hospitals Leuven Leuven Belgium; ^27^ Institute for Medical Biometrics and Epidemiology, University Medical Center Hamburg‐Eppendorf Hamburg Germany; ^28^ Department of Cancer Epidemiology University Cancer Center Hamburg (UCCH), University Medical Center Hamburg‐Eppendorf Hamburg Germany; ^29^ Department of Laboratory Medicine and Pathology Mayo Clinic Rochester MN; ^30^ Cancer Epidemiology and Intelligence Division Cancer Council Victoria Melbourne VIC Australia; ^31^ Genomics Center, Centre Hospitalier Universitaire de Québec Research Center, Laval University Québec City QC Canada; ^32^ Department of Medicine McGill University Montréal QC Canada; ^33^ Division of Clinical Epidemiology Royal Victoria Hospital, McGill University Montréal QC Canada; ^34^ Usher Institute of Population Health Sciences and Informatics, The University of Edinburgh Medical School, Teviot Place Edinburgh Edinburgh United Kingdom; ^35^ Division of Cancer Epidemiology and Genetics National Cancer Institute Bethesda MD; ^36^ Centre for Cancer Genetic Epidemiology Department of Public Health and Primary Care, University of Cambridge, Worts Causeway Cambridge United Kingdom; ^37^ Department of Electron Microscopy/Molecular Pathology The Cyprus Institute of Neurology and Genetics, Nicosia Cyprus Nicosia; ^38^ Department of Medical Epidemiology and Biostatistics Karolinska Institutet Stockholm Sweden; ^39^ Sheffield Institute for Nucleic Acids (SInFoNiA), Department of Oncology and Metabolism, University of Sheffield Sheffield United Kingdom; ^40^ Academic Unit of Pathology Department of Neuroscience, University of Sheffield Sheffield United Kingdom; ^41^ Division of Genetics and Epidemiology The Institute of Cancer Research Sutton, London United Kingdom; ^42^ Division of Breast Cancer Research The Institute of Cancer Research Sutton, London United Kingdom; ^43^ Department of Oncology University of Cambridge, Worts Causeway, Centre for Cancer Genetic Epidemiology Cambridge United Kingdom; ^44^ Division of Psychosocial Research and Epidemiology The Netherlands Cancer Institute–Antoni van Leeuwenhoek Hospital Amsterdam The Netherlands; ^45^ Research Group Genetic Cancer Epidemiology, University Cancer Center Hamburg (UCCH), University Medical Center Hamburg‐Eppendorf Hamburg Germany

**Keywords:** breast cancer, single nucleotide polymorphism, Breast Cancer Association Consortium, gene–environment, interaction

## Abstract

Investigating the most likely causal variants identified by fine‐mapping analyses may improve the power to detect gene–environment interactions. We assessed the interplay between 70 single nucleotide polymorphisms identified by genetic fine‐scale mapping of susceptibility loci and 11 epidemiological breast cancer risk factors in relation to breast cancer. Analyses were conducted on up to 58,573 subjects (26,968 cases and 31,605 controls) from the Breast Cancer Association Consortium, in one of the largest studies of its kind. Analyses were carried out separately for estrogen receptor (ER) positive (ER+) and ER negative (ER–) disease. The Bayesian False Discovery Probability (BFDP) was computed to assess the noteworthiness of the results. Four potential gene–environment interactions were identified as noteworthy (BFDP < 0.80) when assuming a true prior interaction probability of 0.01. The strongest interaction result in relation to overall breast cancer risk was found between *CFLAR*‐rs7558475 and current smoking (OR_int_ = 0.77, 95% CI: 0.67–0.88, *p*_int_ = 1.8 × 10^−4^). The interaction with the strongest statistical evidence was found between 5q14‐rs7707921 and alcohol consumption (OR_int_ =1.36, 95% CI: 1.16–1.59, *p*_int_ = 1.9 × 10^−5^) in relation to ER– disease risk. The remaining two gene–environment interactions were also identified in relation to ER– breast cancer risk and were found between 3p21‐rs6796502 and age at menarche (OR_int_ = 1.26, 95% CI: 1.12–1.43, *p*_int_ =1.8 × 10^−4^) and between 8q23‐rs13267382 and age at first full‐term pregnancy (OR_int_ = 0.89, 95% CI: 0.83–0.95, *p*_int_ = 5.2 × 10^−4^). While these results do not suggest any strong gene–environment interactions, our results may still be useful to inform experimental studies. These may in turn, shed light on the potential interactions observed.

## Abstract 2

**What's new?**

Although it is widely acknowledged that genes and environment may interact to cooperatively modify breast cancer risk, no such interaction is known at the single nucleotide polymorphism (SNP) level. Here, the authors assessed the interplay of 70 SNPs with 11 known breast cancer risk factors in estrogen receptor‐positive and ‐negative disease. Weak interactions were found with individual SNPs and current smoking or alcohol consumption but no strong gene–environment interaction was identified. These data do not support the model of strong modification of genetic cancer risk by environmental factors.

In 1968, MacMahon stated that “In no field are there more complex examples of the gene–environment relationship than in experimental cancer research.”^1^. Following his words and the general opinion that genetic and non‐genetic risk factors do not give rise to the disease solely by acting on independent pathways, several studies have investigated gene–environment interplay in relation to breast cancer risk. Studies of this type are motivated by the fact that the identification of gene–environment interactions in relation to breast cancer could provide insight into the biological mechanisms underlying the disease, allow the distinction of women at high risk from women at lower risk and improve the accuracy of risk prediction models. However, despite large‐scale, international efforts, to date, there are few single nucleotide polymorphisms (SNPs) for which the effect on breast carcinogenesis has been found to be modified by an epidemiological risk factor, and only one of these has been replicated.^2^, ^3^

Several breast cancer risk *loci* that were previously identified in genome‐wide association studies (GWAS) were recently investigated further by genetic fine‐scale mapping in the framework of the Collaborative Oncological Gene‐Environment Study (COGS) using samples from studies participating in the Breast Cancer Association Consortium (BCAC). The SNPs identified in the fine‐mapping studies were further investigated in subsequent functional studies to identify potential causal associations. The consideration of causal variants may improve power to detect gene–environment interplay. However, if no interactions are detected, the weight of evidence against gene–environment interactions for the *locus* in question is strengthened. Additionally, new susceptibility alleles were identified from genotypes generated by imputation using the 1000 Genomes Project reference panel. Therefore, in this analyses, multiplicative gene–environment interaction in relation to breast cancer risk was assessed between 55 potentially causal as well as 15 newly identified SNP alleles, and the following 11 established epidemiological risk factors: age at menarche, oral contraceptive (OC) use, parity, age at first full‐term pregnancy (FTP), number of FTPs, breastfeeding, use of menopausal hormone therapy (MHT), body mass index (BMI), adult height, smoking and alcohol consumption. We also investigated interactions in relation to estrogen receptor (ER) specific breast cancer risk since different disease subtypes may arise through different pathways. The analyses reported in this article are based on the largest, currently available dataset with genetic data and extensive epidemiological information.

## Methods

### Study subjects

Data on subjects of European descent derived from 21 studies participating in the BCAC were pooled. A brief description of each study can be found in Supporting Information Table S1. There were 12 population‐based and 9 non‐population based studies, each contributing at least 200 cases and 200 controls with available SNP data and information on at least one epidemiological risk factor. Subjects were excluded from the gene–environment interaction analyses if they were male, of non‐European origin, a prevalent case or had missing data on age at diagnosis or age at interview, the epidemiological risk factor in question or any of the adjustment variables. Hence, the number of study subjects for each SNP‐risk factor pair varied with the availability of epidemiological data. Analyses were based on between 11,342 subjects (5,445 cases and 5,897 controls) for effect modification by alcohol consumption and 58,573 subjects (26,968 cases and 31,605 controls) for effect modification by number of FTPs. The set of study subjects that were included in at least one gene–environment interaction model comprised 30,000 cases and 34,501 controls. All studies were approved by the relevant ethics committees and informed consent was obtained from all participants.

### SNP selection and genotyping

Genotyping was carried out using an Illumina iSelect array (iCOGS) in the framework of the COGS project (www.nature.com/icogs). With the aim of detecting causal variants, a number of *loci* known to confer breast cancer risk at the time of the design of the iCOGS array were further investigated using fine scale genetic mapping. To improve SNP density, imputation of the respective regions was performed using the March 2012 release of the 1000 Genomes as reference panel. The functional follow‐up work was not carried out centrally for all regions but divided between the different working groups of BCAC and thus the methods used varied somewhat.^4^, ^5^, ^6^, ^7^, ^8^, ^9^, ^10^, ^11^, ^12^, ^13^, ^14^, ^15^, ^16^, ^17^ In addition, imputed genotypes for 15 new susceptibility loci identified through a meta‐analysis of 11 GWAS with genotypes SNPs generated by imputation using the 1000 Genomes Project March 2012 release as the reference panel were used.^5^ A list of the 70 SNPs included in the analyses for this report can be found in Supporting Information Table S2.

### Data filtering

Data from the participating studies were centrally cleaned and harmonized. The information on epidemiological factors was collected at date of reference. In the case–control studies, this was defined as the date of diagnosis for cases and the date of questionnaire for controls, and in the three cohort studies (Cancer Prevention Study II [CPSII], Melbourne Collaborative Cohort Study [MCCS] and UK Breakthrough Generations Study [UKBGS]) information at baseline was used, unless follow‐up information was available. Women who were 54 years or younger at reference were considered pre‐menopausal and women who were older than 54 years at reference were considered postmenopausal. Subjects who were smokers within 1 year before reference date or used MHT within 6 months before reference date were considered to be current smokers and current MHT users. For the case–control studies, BMI was calculated using usual adult weight or weight 1 year before reference (Australian Breast Cancer Family Study, CECILE Breast cancer Study, Gene‐Environment Interaction and Breast Cancer in Germany, kConFab, Kuopio Breast Cancer Project, Mammary Carcinoma Risk Factor Investigation, Mayo Clinic Breast Cancer Study, NCI Polish Breast Cancer Study and Singapore and Sweden Breast Cancer Study), or weight around the age of 20 years (ESTHER Breast Cancer Study, Karolinska Mammography Project for Risk Prediction of Breast Cancer‐prevalent cases and Study of Epidemiology and Risk Factors in Cancer Heredity). For the cohort studies (CPSII, MCCS and UKBGS), BMI was calculated using information from baseline or the latest available questionnaire before diagnosis, if available.

### Statistical analysis

Association analyses of SNP alleles and breast cancer risk were carried out using logistic regression models adjusted for age at reference, study and ethnicity. In all models used in this study, genotyped SNPs were treated as ordinal variables (counts of minor alleles) and imputed SNPs as continuous variables.

The main effects of the epidemiological risk factors were also investigated using logistic regression models adjusted for reference age, study and self‐assessed ethnicity. Heterogeneity across studies was explored by means of Cochrane's Q‐test. The epidemiological variables used in this analyses were categorized as follows: age at menarche (per 2 years), ever use of OC (yes or no), ever parous (yes or no), number of FTPs for parous women (1, 2, 3 and ≥4 FTPs), ever breastfed (yes or no), age at first FTP (per 5 years), adult BMI for pre‐ and postmenopausal women, respectively (per 5 kg/m^2^), adult height (per 5 cm), current use of MHT in the form of estrogen and progesterone or estrogen only (yes or no), lifetime average alcohol intake (per 10 g/day), current smoking (yes or no) and pack‐years of smoking (per 10 pack‐years).

Multiplicative gene–environment interaction was assessed by comparing logistic regression models with and without SNP‐risk factor interaction terms by means of the likelihood ratio test. All models on which this study is based were adjusted for study, reference age and ethnicity, so as to capture genetic population sub‐structure. An interaction term between the epidemiological variable and an indicator for population based study design was included to protect against bias due to the differing selection of study participants in non‐population based *versus* population‐based studies. Interactions of SNPs and epidemiological risk factors were also investigated in relation to ER specific (ER+ or ER–) breast cancer risk, using cases and controls. Furthermore, potentially differential gene–environment interaction according to ER status was assessed in case‐only analyses comparing ER– cases to ER+ cases. The ER‐specific models and the case‐only analyses were adjusted similarly as the interaction models for overall breast cancer risk. To elucidate the results of the interaction analyses, risk association between SNPs and breast cancer was investigated by stratifying on the epidemiological variables.

MHT was sub‐divided into estrogen only and combined (estrogen plus progestogen) therapy and investigated in relation to breast cancer risk using only post‐menopausal women. All statistical models involving MHT use were further adjusted for former MHT use and current use of the MHT preparation (estrogen only or combined) not included in the interaction term. Additionally, interactions of SNPs and BMI for postmenopausal women were assessed in never‐ and former users of MHT only. All risk analyses were carried out using SAS 9.2.

Between‐study heterogeneity of the interaction odds ratio (OR) estimates was investigated using Cochrane's Q‐test and quantified by the ratio of true heterogeneity to the total observed variation, denoted *I^2^*. Heterogeneity was investigated for SNP‐risk factor pairs with an interaction *p* values below the Bonferroni corrected threshold of statistical significance for genetic main effects, computed by dividing the standard threshold of 0.05 by the number of SNPs (0.05/70 > 7 × 10^−4^). Interaction ORs were tested for heterogeneity across studies on basis of interaction *p* values in models of overall or ER specific breast cancer risk, although the latter on the condition that a heterogeneity *p* < 0.05 of ER+ *versus* ER– disease had been observed. Heterogeneity tests were conducted using the R package “rmeta” (version 2.2).

The Bayesian False Discovery Probability (BFDP) was computed to control the number of false‐positive findings and assess the noteworthiness of the results.^18^ The cut‐off for noteworthiness is based on the ratio of the cost of a false non‐discovery to the cost of a false discovery. As suggested in the literature, we set the cost of failing to discover a true association to four times the cost of a falsely reported one, classifying results with a BFDP < 0.8 as noteworthy. The BFDP was calculated for all SNP‐risk factor pairs with an interaction *p* values below the Bonferroni‐corrected threshold given above (*p* < 7 × 10^−4^). The BFDP was computed for two different prior probabilities of this (0.01, 0.001), under the assumption that the probability of observing a true interaction OR inside the interval 0.66–1.5 was 95%. As a complementary measure to the BFDP, we also computed the ABF, which approximates the ratio of the probability of the data given that the null hypothesis is true, to the probability of the data given that the alternative hypothesis is true. The null hypothesis in this case is that the coefficient of the interaction term in the logistic regression model is equal to zero.

## Results

The studies included in the gene–environment interaction analyses are listed in Table 1 together with the number of cases and controls, overall and by ER status. The median time between questionnaire and diagnosis was 3 years in the MCCS cohort, 2 years in the UKBGS cohort and 7 years in the CPSII cohort.

**Table 1:** Participating studies

Study	Full study name	Study design	Country	Cases			Controls
				All	ER–	ER+	
ABCFS	Australian Breast Cancer Family Study	Population‐based	Australia	790	261	456	551
ABCS	Amsterdam Breast Cancer Study	Mixed	Netherlands	1,245	292	800	1,177
BBCC	Bavarian Breast Cancer Cases and Controls	Mixed	Germany	553	86	456	457
BREOGAN	Breast Oncology Galicia Network	Mixed	Spain	1,561	329	1,251	1,423
CECILE	CECILE Breast cancer Study	Population‐based	France	900	128	751	999
CGPS	Copenhagen General Population Study	Mixed	Denmark	2,209	269	1,592	4,506
CPSII	Cancer Prevention Study II	Population‐based	USA	1,655	35	1,205	1,940
ESTHER	ESTHER Breast Cancer Study	Population‐based	Germany	471	98	302	502
GENICA	Gene‐Environment Interaction and Breast Cancer in Germany	Population‐based	Germany	456	114	333	427
KBCP	Kuopio Breast Cancer Project	Population‐based	Finland	411	93	303	251
LMBC	Leuven Multidisciplinary Breast Centre	Mixed	Belgium	2,424	378	2,069	1,045
MARIE	Mammary Carcinoma Risk Factor Investigation	Population‐based	Germany	1,656	371	1,278	1,778
MCBCS	Mayo Clinic Breast Cancer Study	Mixed	USA	1,554	254	1,295	1,893
MCCS	Melbourne Collaborative Cohort Study	Population‐based	Australia	478	117	343	490
MTLGEBCS	Montreal Gene‐Environment Breast Cancer Study	Population‐based	Canada	489	64	421	436
PBCS	NCI Polish Breast Cancer Study	Population‐based	Poland	519		519	424
pKARMA	Karolinska Mammography Project for Risk Prediction of Breast Cancer‐prevalent cases	Mixed	Sweden	2,822	410	2,328	5,469
SASBAC	Singapore and Sweden Breast Cancer Study	Population‐based	Sweden	1,163	144	663	1,378
SBCS	Sheffield Breast Cancer Study	Mixed	UK	751	107	367	848
SEARCH	Study of Epidemiology and Risk Factors in Cancer Heredity	Mixed	UK	7,478	1,119	5,371	8,050
UKBGS	UK Breakthrough Generations Study	Population‐based	UK	415	47	231	457
Total				30,000	4,716	22,334	34,501

The associations between SNP alleles and breast cancer risk in the subset of BCAC studies with risk factor data available were consistent with earlier reports and can be found in Supporting Information Table S3.^4^, ^5^, ^6^, ^7^, ^8^, ^9^, ^10^, ^11^, ^12^, ^13^, ^14^

Main effects of the epidemiological variables on breast cancer risk across studies are presented in Supporting Information Figure 1. These analyses were carried out using only population‐based studies and the results were consistent with what has been reported earlier in the literature.^3^, ^19^, ^20^, ^21^, ^22^, ^23^, ^24^, ^25^, ^26^, ^27^, ^28^, ^29^, ^30^ Current use of OC, MHT use (E only, as well as E + P), alcohol consumption, height as well as never having breastfed (*vs*. ever having breastfed) were all factors that showed an increased risk of breast cancer. A reduction in risk was observed for older age at menarche, ever being parous, number of FTPs and high BMI for pre‐menopausal women. For current smoking and pack‐years of smoking, no significant association with breast cancer risk was detected.

The complete results from the interaction analyses, showing the risk association between SNPs and breast cancer across categories of the epidemiological variables, are presented in Supporting Information Table S4. We identified four SNP‐risk factor pairs with at least one interaction *p* < 7 × 10^−4^ in relation to overall, ER+ or ER– breast cancer risk, as presented in Table 2. All of these interactions were classified as noteworthy (BFDP < 0.8) assuming a prior probability of true interaction of 0.01 but no result remained noteworthy at the 0.001 level (Table 3).

**Table 2:** SNP‐risk factor pairs with interaction *p* < 7 × 10^−4^, overall and by ER status across categories of epidemiological risk factors

SNP/risk factor	Stratum	Cases/controls	Overall			ER+		ER–		*P*_case‐only_
			OR (95% CI)	*p*_interaction_		OR (95% CI)	*p*_interaction_	OR (95% CI)	*p*_interaction_	
*CFLAR*‐rs7558475/current smoking	No	7,698/8,835	0.99 (0.91–1.08)			1.00 (0.91–1.09)		0.96 (0.81–1.14)		
	Yes	1,375/1,519	0.76 (0.66–0.88)			0.78 (0.66–0.91)		0.91 (0.63–1.31)		
	All	9,073/10,354	0.96 (0.88–1.04)		1.8 × 10^−4^	0.96 (0.88–1.05)	0.0014	0.96 (0.82–1.12)	0.75	0.48
5q14‐rs7707921/alcohol consumption	<20 g/day	4,904/5,411	1.16 (1.08–1.24)			1.18 (1.10–1.27)		1.07 (0.92–1.24)		
	≥20 g/day	481/541	1.16 (0.94–1.43)			1.08 (0.86–1.36)		2.59 (1.45–4.62)		
	All	5,385/5,952	1.16 (1.09–1.23)		0.70	1.17 (1.09–1.25)	0.79	1.15 (0.99–1.32)	1.9 × 10^−5^	6.7 × 10^−6^
3p21‐rs6796502/age at menarche	≤11 years	3,350/3,609	0.73 (0.65–0.83)			0.75 (0.65–0.86)		0.70 (0.54–0.90)		
	12–13 years	9,503/10,893	0.93 (0.86–0.99)			0.93 (0.86–1.00)		0.88 (0.76–1.02)		
	≥14 years	7,294/8,864	0.95 (0.88–1.03)			0.92 (0.84–1.01)		1.16 (0.99–1.34)		
	All	20,147/23,366	0.90 (0.86–0.95)		0.94	0.89 (0.85–0.94)	0.73	0.94 (0.86–1.04)	1.8 × 10^−4^	0.53
8q23‐rs13267382/age at first FTP	<20 years	2,085/1,830	1.01 (0.92–1.11)			1.00 (0.90–1.11)		1.12 (0.94–1.33)		
	20–24 years	6,944/8,246	0.92 (0.88–0.97)			0.91 (0.86–0.96)		1.00 (0.91–1.11)		
	25–29 years	5,388/6,877	0.97 (0.92–1.02)			0.98 (0.92–1.03)		0.91 (0.81–1.02)		
	≥30 years	2,965/3,555	0.92 (0.85–0.99)			0.94 (0.87–1.02)		0.79 (0.68–0.91)		
	All	17,382/20,508	0.94 (0.92–0.97)		0.47	0.95 (0.91–0.98)	0.98	0.95 (0.89–1.01)	5.2 × 10^−4^	0.99

**Table 3:** BFDPs for SNP‐risk factor pairs with interaction *p* < 7 × 10^−4^

Breast cancer subtype	SNP/risk factor	OR_interaction_ (95%CI)	BFDP^1^, prior probability of true interaction		ABF^2^
			0.01	0.001	
Overall	*CFLAR*‐rs7558475/current smoking	0.77 (0.67–0.88)	0.40	0.87	0.007
ER–	5q14‐rs7707921/alcohol	1.36 (1.16–1.59)	0.33	0.83	0.005
ER–	3p21‐rs6796502/age at menarche	1.26 (1.12–1.43)	0.49	0.91	0.010
ER–	8q23‐rs13267382/age at first FTP	0.89 (0.83–0.95)	0.61	0.94	0.016

The BFDP was calculated assuming that the true interaction OR is between 0.66 and 1.50. ^2^ABF is an approximation of the rate of the probability of the data given the null to the probability of the data given the alternative hypothesis.

First evidence of an interaction in relation to overall disease risk was noted between the variant *CFLAR*‐rs7558475 and current smoking (OR_int_ = 0.77, 95% CI: 0.67–0.88, *p*_int_ = 1.8 × 10^−4^). This result was considered noteworthy (BFDP = 0.40) assuming a prior probability of true interaction of 0.01 and the approximated Bayes factor (ABF) = 0.007 indicated that the data were almost 140 times more likely given the alternative hypothesis than given the null. Breast cancer risk was reduced for current smokers carrying the minor allele (G) (OR_per‐allele_ = 0.76, 95% CI: 0.66–0.88, *p* = 2.2 × 10^−4^) compared to that of non‐smoker carriers (OR_per‐allele_ = 0.99, 95% CI: 0.91–1.08, *p* = 0.9) where no risk association was observed. When comparing ER– cases to ER+ cases, the results did not indicate any effect heterogeneity (*p*_het_ = 0.48). There was no strong evidence of interaction, neither with respect to ER+ risk (*p*_int_ = 0.0014) nor with respect to ER– risk (*p*_int_ = 0.75).

The most promising result of the gene–environment interaction analyses in terms of noteworthiness was considered noteworthy at the 0.01 probability level and was noted between the variant 5q14‐rs7707921 located in an intron of the autophagy related 10 (*ATG10*) gene, and alcohol consumption (OR_int_ = 1.36, 95% CI: 1.16–1.59, *p*_int_ = 1.9 × 10^−5^) in relation to ER– breast cancer. This result had the lowest BFDP = 0.33, and conditioning on the alternative, the data were about 200 times more likely as compared to conditioning on the null (ABF = 0.005). Carriers of the minor allele (T) of rs7707921 had an increased risk of ER– breast cancer if they consumed >20 g of alcohol per day (OR_per‐allele_ = 2.56, 95% CI: 1.45–4.62, *p* = 0.001), but not if they consumed <20 g of alcohol per day (OR_per‐allele_ = 1.07, 95% CI: 0.92–1.24, *p* = 0.36). A strong effect heterogeneity was detected when comparing ER– cases to ER+ cases (*p*_het_ = 6.7 × 10^−6^). Together with the absence of interaction in relation to ER+ disease (*p*_int_ = 0.79) and overall breast cancer risk (*p*_int_ = 0.70), this indicated that the interaction might be specific to ER– disease.

In addition, indications of two further interactions were noted in relation to ER– disease risk. One of these was between 3p21‐rs6796502 and age at menarche (OR_int_ = 1.26, 95% CI: 1.12–1.43, *p*_int_ =1.8 × 10^−4^) which had BFDP = 0.49, and of which the ABF (ABF = 0.010) implied that the data were 100 times more likely under the alternative hypothesis than under the null. Carriers of the minor allele (A) of 3p21‐rs6796502 who experienced their menarche no later than the age of 11 years had a reduced risk of ER– breast cancer (OR_per‐allele_ = 0.70, 95% CI: 0.54–0.90, *p* = 0.006), whereas there was no association with disease risk of the genetic variant for women who had their menarche between the age of 12 and 13 years (OR_per‐allele_ = 0.88, 95% CI: 0.76–1.02, *p* = 0.08), or after the age of 14 years (OR_per‐allele_ = 1.16, 95% CI: 0.99–1.34, *p* = 0.06). While the observed interaction was in relation to ER– disease risk, no effect heterogeneity was detected when comparing ER– and ER+ cases (*p*_het_ = 0.53) nor was there any indication of any interaction in relation to overall breast cancer risk (*p*_int_ = 0.94). Hence, it is not possible to conclude that the observed interaction is specific for ER– disease.

Finally, an indication of a gene–environment interaction was found between 8q23‐rs13267382 and age at first FTP (OR_int_ = 0.89, 95% CI: 0.83–0.95, *p*_int_ = 5.2 × 10^−4^) in relation to ER– disease risk. This interaction had BFDP = 0.61 assuming a true, prior interaction probability of 0.01, and ABF = 0.016, indicating that the data are about 60 times more likely conditioning on the alternative than on the null. There was no interaction observed in relation to disease risk, when considering ER+ breast cancer (*p*_int_ = 0.98), or overall breast disease risk (*p*_int_ = 0.47), and no effect heterogeneity was found when comparing the risk of ER– and ER+ breast cancer (*p*_het_ = 0.99). Our findings indicated a modest reduction in ER– breast cancer risk for minor allele (A) carriers who were aged 30 or above at their first FTP (OR_per‐allele_ = 0.79, 95% CI: 0.68–0.91, *p* = 0.001), whereas for women who had their first child at younger ages the allele had no effect on risk.

## Discussion

From the analyses presented in this work, four SNP‐risk factor pairs were identified, for which *p*_int_ < 7 × 10^−4^, and all of the interactions were considered noteworthy (BFDP < 0.8) assuming a true prior interaction probability of 0.01. One of the results was detected in relation to overall breast cancer risk, while the three remaining results appeared to be specific for ER– disease.

The strongest gene–environment interaction in relation to overall breast cancer risk was noted between rs7558475 located in the *CASP8* and *FADD* like apoptosis regulator (*CFLAR*) gene and current smoking (*p*_int_ = 1.8 × 10^−4^). The protein product of *CFLAR* regulates apoptosis, thus it is possible that *CFLAR* genetic variants affect response to DNA damage caused by tobacco associated carcinogens and therefore modify breast cancer risk conferred by smoking. However, although rs7558475 is located in a *CFLAR* enhancer region, reports from recent functional studies and expression quantitative trait locus (eQTL) analyses did not provide any convincing evidence regarding functionality.^6^, ^31^ Hence, further work is required to understand possible biological mechanism related to the observed interaction.

The strongest statistical evidence of interaction was found in relation to ER– breast cancer risk and was noted between an intron variant 5q14‐rs7707921 in the autophagy related 10 (*ATG10*) gene, and alcohol consumption (*p*_int_ =1.9 × 10^−5^). Autophagy, which is considered a survival mechanism of the cell, may act as a tumor suppressor but also influence cell survival by promoting tumor growth, and has been suggested as a target in cancer therapy.^32^ It has been reported that autophagy could have a protective effect on esophageal epithelial cells responding to ethanol‐induced oxidative stress.^33^ Also, while ethanol promotes oxidative stress in cancer associated fibroblasts, it has been reported to induce autophagy resistance in epithelial cells.^34^ Given the above information, it is conceivable that alcohol consumption could influence the effect on breast cancer risk of an autophagy‐related polymorphism. However the biological mechanism needs to be further investigated. The position of the variant *ATG10*‐rs7707921 does not coincide with any strong regulatory elements. The eQTL analyses carried out within the framework of BCAC showed a strong association between the T allele of rs7707921 and expression of the ribosomal protein S23 gene (*RPS23*) in breast tissue as well as a moderate association between the allele and expression of the ATPase, H+ transporting, lysosomal accessory protein 1‐like (*ATP6AP1L*) gene.^5^ The *RPS23* gene encodes a ribosomal protein and the *ATP6AP1L* is also protein coding but the genes have not yet been implicated in ER– breast cancer risk and their expression levels have not been assessed in relation to alcohol consumption or oxidative stress. Further work is thus needed to understand how the protein products of these genes could interact with alcohol consumption to modify the risk association of rs7707921 with ER– breast cancer.

Furthermore, we found an indication of a possible interaction between 3p21‐rs6796502 and age at menarche (*p*_int_ = 1.8 × 10^−4^) in relation to ER– breast cancer. Our results suggest that the reduced risk of ER– breast tumors for carriers of the A‐allele are modified for women with late age at menarche ≥ 14 years. However, according to a recent functional study, the SNP is not located in the vicinity of any genes or enhancer regions in mammary cell lines, nor are there any significant results available from eQTL analyses.^5^ In addition, no significant effect heterogeneity was found when comparing the interaction between ER– and ER+ cases to support that the result could to be specific to ER– disease. It is thus necessary to first confirm this interaction with further data before attempting any biological explanation.

The interaction observed between the intron variant 8q23‐rs13267382 of the long intergenic non protein coding RNA 536 gene (*LINC00536*) and age at first FTP (*p*_int_ =2.6 × 10^−4^) suggests that the variant is associated with a reduced risk of ER– breast cancer with older age at first FTP, whereby the association was statistically significant for women who were at least 30 years of age at their first FTP. Overall, this variant was not reported to be associated with ER– disease risk,^5^ which is confirmed in the current report. Neither this SNP nor any variants in high linkage disequilibrium with it are positioned in the vicinity of any regulatory genomic feature. As for the interaction with 3p21‐rs6796502, there was not clear evidence for this interaction to be specific to ER– disease. Therefore, further data are required to confirm this interaction.

This work is subject to a number of limitations. First, despite central harmonization of the data, substantial heterogeneity was observed in the risk estimates of the epidemiological risk factors across studies, which brought about the inclusion of a product term of study design and epidemiological variable in the interaction models, and the quantification of epidemiological main effects based on the population based studies. Second, the study population consisted predominantly of case‐control studies (only three cohort studies), which are known to be prone to selection bias and recall bias, as well as associated misclassification of risk factors. However, gene–environment interaction estimates were similar in the overall study population compared to the subset of population based studies (data not shown). Misclassification of epidemiological risk factors is known to reduce the power to detect interactions, rather than increasing the probability of false‐positive findings.^35^ Hence, this study is more likely to be subject to limited power than to spurious gene–environment interactions. Also, our findings are based on study participants of Caucasian origin so that they may not be generalizable to other populations. For the ER specific risk analyses, in particular in the subgroup of ER– cases (*N* = 4,662), the power was diminished due to the reduced sample size.

However, this study also has several strengths. To begin with, the interaction analyses are based on the largest dataset presently available. The four indicated interactions were based on 11,337 subjects (5,385 cases and 5,952 controls) in analyses with respect to alcohol consumption, and 19,427 subjects (9,073 cases and 10,354 controls) for current smoking, as well as 43,513 subjects (20,147 cases and 23,366 controls) in the analyses of age at menarche and 37,819 subjects (17,382 cases and 20,508 controls) in the analyses of age at first FTP.

Taken together, the results presented in this report are not in line with the existence of strong modification of the allelic effects on breast cancer risk by the epidemiological risk factors investigated. However, the results presented in this report contribute to the global body of knowledge on gene–environment interactions by generating hypotheses, thereby providing guidance for future functional studies and large scale replication studies.

## Supporting information

Supporting Information Figure 1.

Supporting Information Table 1.

Supporting Information Table 2.

Supporting Information Table 3.

Supporting Information Table 4.

## References

[1] MacMahon B. Gene‐environment interactions in human disease. J PsychiatrRes 1968;6:393–402.

[2] Rudolph A , Chang‐Claude J , Schmidt MK. Gene‐environment interaction and risk of breast cancer. Br J Cancer 2016;114:125–33. 26757262 10.1038/bjc.2015.439 PMC4815812

[3] Milne RL , Gaudet MM , Spurdle AB , et al. Assessing interactions between the associations of common genetic susceptibility variants, reproductive history and body mass index with breast cancer risk in the breast cancer association consortium: a combined case‐control study . Breast Cancer Res 2010;12:R110. 21194473 10.1186/bcr2797 PMC3046455

[4] Darabi H , McCue K , Beesley J , et al. Polymorphisms in a putative enhancer at the 10q21.2 breast cancer risk locus regulate NRBF2 expression. Am J Hum Genet 2015;97:22–34. 26073781 10.1016/j.ajhg.2015.05.002 PMC4572510

[5] Michailidou K , Beesley J , Lindstrom S , et al. Genome‐wide association analysis of more than 120,000 individuals identifies 15 new susceptibility loci for breast cancer. Nat Genet 2015;47:373–80. 25751625 10.1038/ng.3242 PMC4549775

[6] Lin WY , Camp NJ , Ghoussaini M , et al. Identification and characterization of novel associations in the CASP8/ALS2CR12 region on chromosome 2 with breast cancer risk. Hum Mol Genet 2015;24:285–98. 25168388 10.1093/hmg/ddu431 PMC4334820

[7] Ghoussaini M , Edwards SL , Michailidou K , et al. Evidence that breast cancer risk at the 2q35 locus is mediated through IGFBP5 regulation. Nat Commun 2014;4:4999 25248036 10.1038/ncomms5999 PMC4321900

[8] Bojesen SE , Pooley KA , Johnatty SE , et al. Multiple independent variants at the TERT locus are associated with telomere length and risks of breast and ovarian cancer. Nat Genet 2013;45:371–84. 84e1–2. 23535731 10.1038/ng.2566 PMC3670748

[9] Milne RL , Burwinkel B , Michailidou K , et al. Common non‐synonymous SNPs associated with breast cancer susceptibility: findings from the Breast Cancer Association Consortium. Hum Mol Genet 2014;23:6096–111. 24943594 10.1093/hmg/ddu311 PMC4204770

[10] Meyer KB , O'reilly M , Michailidou K , et al. Fine‐scale mapping of the FGFR2 breast cancer risk locus: putative functional variants differentially bind FOXA1 and E2F1. Am J Hum Genet 2013;93:1046–60. 24290378 10.1016/j.ajhg.2013.10.026 PMC3852923

[11] French JD , Ghoussaini M , Edwards SL , et al. Functional variants at the 11q13 risk locus for breast cancer regulate cyclin D1 expression through long‐range enhancers. Am J Hum Genet 2013;92:489–503. 23540573 10.1016/j.ajhg.2013.01.002 PMC3617380

[12] Guo X , Long J , Zeng C , et al. Fine‐scale mapping of the 4q24 locus identifies two independent loci associated with breast cancer risk. Cancer Epidemiol Biomarkers Prev 2015;24:1680–91. 26354892 10.1158/1055-9965.EPI-15-0363 PMC4633342

[13] Glubb DM , Maranian MJ , Michailidou K , et al. Fine‐scale mapping of the 5q11.2 breast cancer locus reveals at least three independent risk variants regulating MAP3K1. Am J Hum Genet 2015;96:5–20. 25529635 10.1016/j.ajhg.2014.11.009 PMC4289692

[14] Orr N , Dudbridge F , Dryden N , et al. Fine‐mapping identifies two additional breast cancer susceptibility loci at 9q31.2. Hum Mol Genet 2015;24:2966–84. 25652398 10.1093/hmg/ddv035 PMC4406292

[15] Dunning AM , Michailidou K , Kuchenbaecker KB , et al. Breast cancer risk variants at 6q25 display different phenotype associations and regulate ESR1, RMND1 and CCDC170. Nat Genet 2016;48:374–86. 26928228 10.1038/ng.3521 PMC4938803

[16] Shi J , Zhang Y , Zheng W , et al. Fine‐scale mapping of 8q24 locus identifies multiple independent risk variants for breast cancer. Int J Cancer 2016;139:1303–1317. 27087578 10.1002/ijc.30150 PMC5110427

[17] Horne HN , Chung CC , Zhang H , et al. Fine‐mapping of the 1p11.2 breast cancer susceptibility locus. PloS One 2016;11:e0160316 27556229 10.1371/journal.pone.0160316 PMC4996485

[18] Wakefield J. A Bayesian measure of the probability of false discovery in genetic epidemiology studies. Am J Hum Genet 2007;81:208–27. 17668372 10.1086/519024 PMC1950810

[19] Rudolph A , Milne RL , Truong T , et al. Investigation of gene‐environment interactions between 47 newly identified breast cancer susceptibility loci and environmental risk factors. Int J Cancer 2015;136:E685–96. 25227710 10.1002/ijc.29188 PMC4289418

[20] Nickels S , Truong T , Hein R , et al. Evidence of gene‐environment interactions between common breast cancer susceptibility loci and established environmental risk factors. PLoS Genet 2013;9: e1003284. 23544014 10.1371/journal.pgen.1003284 PMC3609648

[21] Barrdahl M , Canzian F , Joshi AD , et al. Post‐GWAS gene‐environment interplay in breast cancer: results from the Breast and Prostate Cancer Cohort Consortium and a meta‐analysis on 79,000 women. Hum Mol Genet 2014;23:5260–70. 24895409 10.1093/hmg/ddu223 PMC4159150

[22] Campa D , Kaaks R , Le Marchand L , et al. Interactions between genetic variants and breast cancer risk factors in the breast and prostate cancer cohort consortium. J Natl Cancer Inst 2011;103:1252–63. 21791674 10.1093/jnci/djr265 PMC3156803

[23] Baer HJ , Rich‐Edwards JW , Colditz GA , et al. Adult height, age at attained height, and incidence of breast cancer in premenopausal women. Int J Cancer 2006;119:2231–5. 16823843 10.1002/ijc.22096

[24] Bassuk SS , Manson JE. Oral contraceptives and menopausal hormone therapy: relative and attributable risks of cardiovascular disease, cancer, and other health outcomes. Ann Epidemiol 2014;25:193–200. 25534509 10.1016/j.annepidem.2014.11.004 PMC4389282

[25] Beaber EF , Malone KE , Tang MT , et al. Oral contraceptives and breast cancer risk overall and by molecular subtype among young women. *Cancer* . Epidemiol Biomarker Prev 2014;23:755–64. 10.1158/1055-9965.EPI-13-0944 PMC4032363 24633144

[26] Beral V , Million Women Study C. Breast cancer and hormone‐replacement therapy in the Million Women Study. Lancet 2003;362:419–27. 12927427 10.1016/s0140-6736(03)14065-2

[27] Calle EE , Feigelson HS , Hildebrand JS , et al. Postmenopausal hormone use and breast cancer associations differ by hormone regimen and histologic subtype. Cancer 2009;115:936–45. 19156895 10.1002/cncr.24101

[28] Collaborative Group on Hormonal Factors in Breast C . Menarche, menopause, and breast cancer risk: individual participant meta‐analysis, including 118 964 women with breast cancer from 117 epidemiological studies. Lancet Oncol 2012;13:1141–51. 23084519 10.1016/S1470-2045(12)70425-4 PMC3488186

[29] Fagherazzi G , Vilier A , Boutron‐Ruault MC , et al. Alcohol consumption and breast cancer risk subtypes in the E3N‐EPIC cohort. Eur J Cancer Prev 2015;24:209–14. 24743350 10.1097/CEJ.0000000000000031

[30] Kelsey JL , Gammon MD , John EM. Reproductive factors and breast cancer. Epidemiol Rev 1993;15:36–47. 8405211 10.1093/oxfordjournals.epirev.a036115

[31] Hnisz D , Abraham BJ , Lee TI , et al. Super‐enhancers in the control of cell identity and disease. Cell 2013;155:934–47. 24119843 10.1016/j.cell.2013.09.053 PMC3841062

[32] Sharma N , Thomas S , Golden EB , et al. Inhibition of autophagy and induction of breast cancer cell death by mefloquine, an antimalarial agent. Cancer Lett 2012;326:143–54. 22863539 10.1016/j.canlet.2012.07.029

[33] Tanaka K , Whelan KA , Chandramouleeswaran PM , et al. ALDH2 modulates autophagy flux to regulate acetaldehyde‐mediated toxicity thresholds. Am J Cancer Res 2016;6:781–96. 27186430 PMC4859883

[34] Sanchez‐Alvarez R , Martinez‐Outschoorn UE , Lin Z , et al. Ethanol exposure induces the cancer‐associated fibroblast phenotype and lethal tumor metabolism: implications for breast cancer prevention. Cell Cycle 2013;12:289–301. 23257780 10.4161/cc.23109 PMC3575458

[35] Garcia‐Closas M , Rothman N , Lubin J. Misclassification in case‐control studies of gene‐environment interactions: assessment of bias and sample size. Cancer Epidemiol Biomarker Prev 1999;8:1043–50. 10613335

