# Supplementary material for: Gene–environment interactions involving functional variants: Results from the Breast Cancer Association Consortium
Source: Int J Cancer. 2017 Aug 11;141(9):1830–40. doi: 10.1002/ijc.30859 (PMC5601244; doi:10.1002/ijc.30859)

**Supplementary Figure 1.** Forest plots of odds ratios and 95% confidence intervals of population-based studies for risk associations between established epidemiological risk factors and breast cancer:

1. **Age at menarche (per 2 years)**


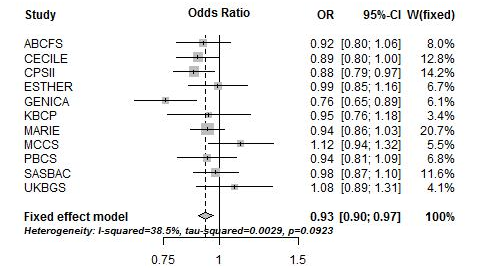


1. **Ever use of oral contraceptives (yes/no)**


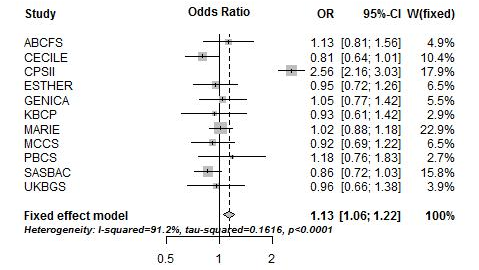


1. **Ever had a full-term pregnancy (yes/no)**


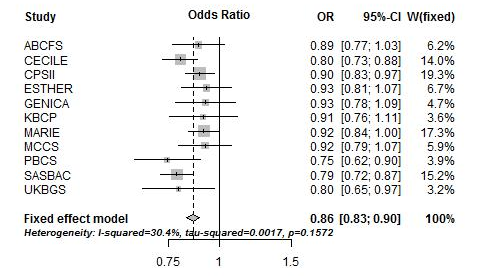


1. **Age at first full-term pregnancy (per 5 years)**


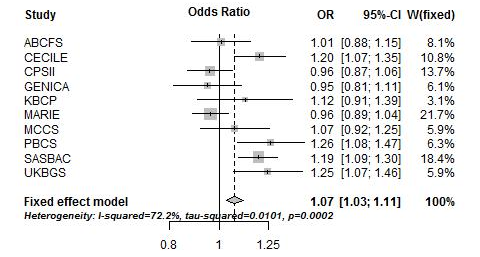


1. **Number of full-term pregnancies (among parous, per pregnancy)**


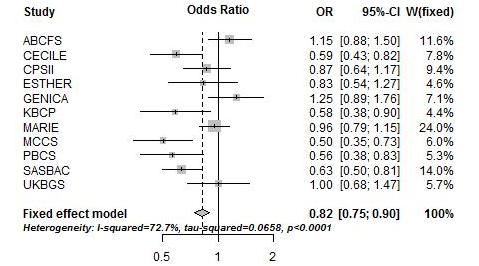


1. **Ever breastfed (no/yes)**


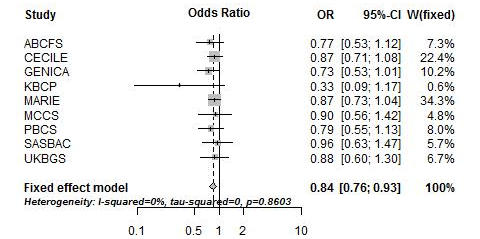


1. **Current use of any menopausal hormone therapy (yes/no)**


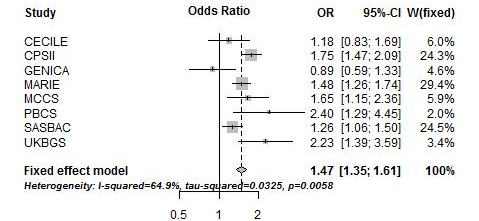


1. **Current use of estrogen-only therapy (yes/no)**


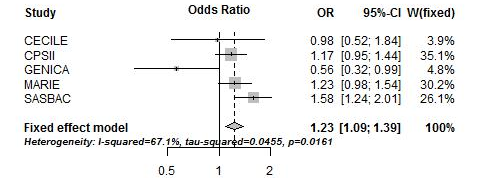


1. **Current use of combined estrogen-progesterone therapy (yes/no)**


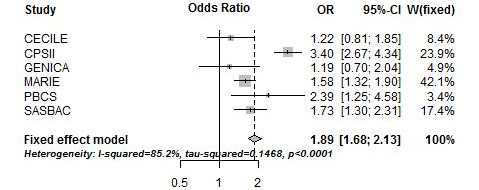


1. **BMI, premenopausal women (per 5 kg/m^2^)**


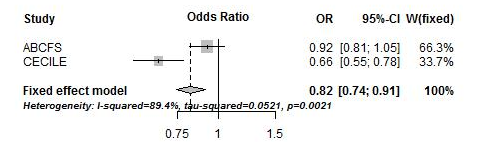


1. **BMI, postmenopausal women2 (per 5 kg/m^2^)**


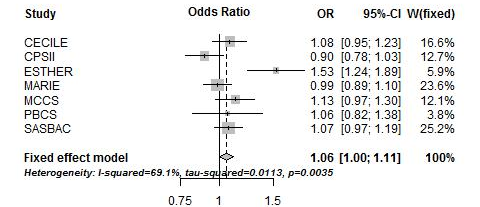


1. **Adult height (per 5 cm)**


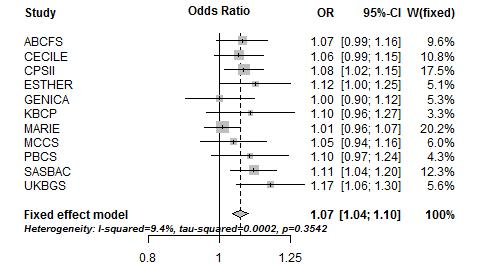


1. **Current smoking (yes/no)**


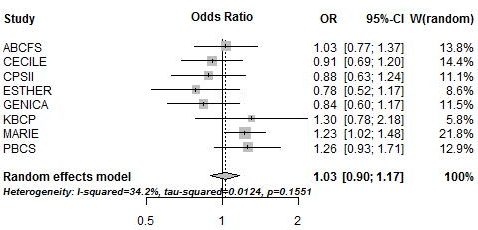


1. **Pack-years of smoking (per 10 pack-years)**


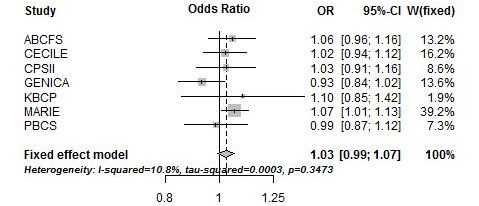


1. **Lifetime intake of alcohol (per 10 g/day)**


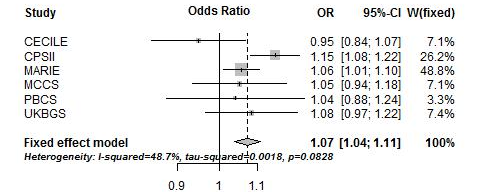

Supplement: Supplementary file 1 — Supporting Information Figure 1. [file IJC-141-1830-s001.docx]
