# Supplementary material for: Gene–environment interactions involving functional variants: Results from the Breast Cancer Association Consortium
Source: Int J Cancer. 2017 Aug 11;141(9):1830–40. doi: 10.1002/ijc.30859 (PMC5601244; doi:10.1002/ijc.30859)
Supplement: Supplementary file 2 — Supporting Information Table 1. [file IJC-141-1830-s002.doc]

**Supplementary Table 1.** Description of BCAC studies included in the analysis of gene-environment interaction

| **Study Acronym** | **Study Name**  **[Reference]** | **Country** | **Recruitment base** | |
| --- | --- | --- | --- | --- |
|  |  |  | **Cases** | **Controls** |
|  |  |  |  |  |
| ABCFS | Australian Breast Cancer Family Study | Australia | All cases diagnosed < age 40 plus a random sample of those diagnosed ages 40-59 from cancer registries in Victoria and New South Wales, plus a limited number diagnosed aged 60-69; cases living in Melbourne recruited from 1992-99 and in Sydney from 1993-98. | Identified from the electoral rolls in Melbourne from 1992-98 and Sydney from 1993-99. Frequency matched to cases by age in 5 year categories. |
| ABCS | Amsterdam Breast Cancer Study | Netherlands | All cases (operable, invasive breast cancer) aged <50 and diagnosed from 1974-1994 in 4 Dutch hospitals. [Familial non-BRCA1/2 cases <50 from Clinical Genetic Centre in the Netherlands Cancer Institute] REMARK: these familial cases were only included in phase I/II. | Random women <50 years of age at baseline from 2 population-based prospective studies - the Monitoring Project on Cardiovascular Risk Factors (1987-1991) and the Monitoring Project on Chronic Disease Risk Factors (1993-1997). Controls are selected from the same catchment area as cases. |
| BBCC | Bavarian Breast Cancer Cases and Controls | Germany | Consecutive, unselected cases with invasive breast cancer recruited at the University Breast Centre, Franconia in Northern Bavaria from 2002-2006. | Healthy women aged 55 or older with no diagnosis of cancer. Invited by a newspaper advertisement in Northern Bavaria in 2002-2006. |
| BREOGAN | Breast Oncology Galicia Network | Spain | Two groups of cases: (1) A population-based study conducted since 2004 in two cities in Galicia, Spain (Vigo and Santiago) covering approx. 700,000 inhabitants. The study currently includes 1,500 breast cancer cases with blood plus risk factor questionnaire. (2) 365 consecutive breast cancer diagnosed at the Clinical University Hospital of Santiago, (CHUS) between 1991 and 2005 with questionnaire, follow-up, DNA, fresh frozen and paraffin embedded tissue. | Random sample of individuals from the same base population selected from the Galician Public Health Service (SERGAS) registry database, which covers more than 95% of the region's population. Populations selected corresponded to primary healthcare centers in the health areas.. Recruitment began in 1993. |
| CECILE | CECILE Breast cancer study | France | All cases diagnosed with breast cancer in 2005-2007 among women <75 years of age residing in the *départements* of Ille-et-Vilaine and Côte d'Or . Cases were recruited from the main cancer treatment center (Centre Eugène-Marquis in Rennes and Centre Georges-François-Leclerc in Dijon) and from other private or public hospitals in each area. | General population control women residing in the same areas as the cases (Ille-et-Vilaine and Côte d’Or). Controls were frequency-matched to the cases by 5-year age groups. They were recruited in 2005-2007 using a random digit dialling procedure and quotas by socioeconomic status to reflect the distribution by SES of the population in each area. |
| CGPS | Copenhagen General Population Study | Denmark | Consecutive, incident cases from one hospital with centralized care for a population of 400,000 women in Copenhagen (2001-present). | Community controls residing in the same region as cases and with no history of breast cancer were identified from the Copenhagen General Population Study recruited 2003-2007. All controls were known to still be breast cancer-free at the end of 2007. |
| CPS2 | Cancer Prevention Study-II Nutrition Cohort | USA | Breast cancer cases from the CPS-II Nutrition cohort were diagnosed between 1992 and 2009. The cases self-reported their diagnoses on biennial questionnaires and the diagnoses were then validated by medical records or state cancer registries. The participants in the Nutrition cohort were a subset of participants who lived in 21 U.S. states and were in the original CPS-II Cohort. The CPS-II Cohort participants were recruited in 1982 by American Cancer Society volunteers who went door-to-door to family, friends, and neighbours. | Controls were matched to cases using a 1:1 matching based on race, birthdate (+/- 6 months), and draw date (+/- 6 months). The participants in the Nutrition cohort were a subset of participants who lived in 21 U.S. states and were in the original CPS-II Cohort. The CPS-II Cohort participants were recruited in 1982 by American Cancer Society volunteers who went door-to-door to family, friends, and neighbours. |
| ESTHER | ESTHER Breast Cancer Study | Germany | Statewide recruitment of breast cancer cases in all hospitals in Saarland/Germany in 2001-2003 | Statewide recruitment of participants of a routine health check-up in Saarland/Germany in 2000-2002. A stratified random sample, matched to the cases by five year age groups, was selected as controls. |
| GENICA | Gene Environment Interaction & Breast Cancer in Germany | Germany | Incident breast cancer cases enrolled between 2000 and 2004 from the Greater Bonn area; all enrolled within 6 months of diagnosis | Selected from population registries from 31 communities in the greater Bonn area; matched to cases in 5-year age classes between 2001 and 2004 |
| KBCP | Kuopio Breast Cancer Project | Finland | Women seen at Kuopio University Hospital between 1990-1995 because of a breast lump, mammographic abnormality, or other breast symptom and who were found to have breast cancer. | Age and long-term area-of-residence matched controls selected from the National Population Register and interviewed in parallel with the cases |
| LMBC | Leuven Multidisciplinary Breast Centre | Belgium | All patients diagnosed with breast cancer and seen in the Multidisciplinary Breast Center in Leuven (University Hospitals Leuven) since June 2007 plus retrospective collection of cases diagnosed since 2000. | Healthy controls (blood donors) collected at the Red Cross and located in University Hospitals Leuven (2007 - 2008) |
| MARIE | Mammary Carcinoma Risk Factor Investigation | Germany | Incident cases diagnosed from 2001-2005 in the study region Hamburg in Northern Germany, and from 2002-2005 in the study region Rhein-Neckar-Karlsruhe in Southern Germany. | 2 controls per case were randomly drawn from population registries and frequency matched by birth year and study region to the case. Controls were recruited from 2002 to 2006. |
| MCBCS | Mayo Clinic Breast Cancer Study | USA | Incident cases residing in 6 states (MN, WI, IA, IL, ND, SD) seen at the Mayo Clinic in Rochester, MN from 2002-2010. | Women without cancer presenting for general medical examination at the Mayo Clinic from 2002-2010; frequency matched to cases on age, ethnicity and county/state. |
| MCCS | Melbourne Collaborative Cohort Study | Australia | Incident cases from the cohort of 24,469 women, diagnosed during the follow-up from baseline (1990-1994) to 2008. Cases and their vital status were ascertained through the Victorian Cancer Registry (VCR) and the Australian Institute of Health and Welfare (AIHW), including the National Death Index and the Australian Cancer Database. | Random sample of the initial cohort. |
| MTLGEBCS | Montreal Gene-Environment Breast Cancer Study | Canada | All cases are postmenopausal women (47-75 years) living in Montreal with a primary invasive breast cancer and with no previous occurrence of any type of cancer. All cases were identified from 2007 to 2010 in 15 of 16 Montreal hospitals that treat breast cancer. | All controls are postmenopausal women (47-75 years) living in Montreal with no personal history of cancer. All controls were identified using the Quebec provincial electoral list from 2007-2010. The electoral list has close to 100% coverage of Canadian citizens living in the Province. |
| PBCS | NCI Polish Breast Cancer Study | Poland | Incident cases identified through a rapid identification system in participating hospitals covering ~ 90% of all eligible cases, and cancer registries in Warsaw and Łódź covering 100% of all eligible cases (2000-2003). | Randomly selected from population lists of all residents of Poland from 2000-2003, stratified and frequency matched to cases on city and age in 5-year categories. |
| pKARMA | Karolinska Mammography Project for Risk Prediction of Breast Cancer - prevalent cases | Sweden | Incident cases from Jan 2001 – Dec 2008 from the Stockholm/Gotland area. Identified through the Stockholm breast cancer registry. | Unmatched participants of the KARMA mammography screening study recruited between 2010 and 2011 from Helsingborg and Stockholm. |
| SASBAC | Singapore and Sweden Breast Cancer Study | Sweden | Incident cases from 1993 to 1995 identified via the 6 regional cancer registries in Sweden, to which reporting is mandatory. | Controls were randomly selected from the total population registry in 5-year age groups to match the expected age-frequency distribution among cases. Patients and controls were recruited from 1993 through 1995. |
| SBCS | Sheffield Breast Cancer Study | UK | Women with breast cancer recruited in 1998-2005 at surgical outpatient clinics at the Royal Hallamshire Hospital, Sheffield. | Unselected women attending the Sheffield Mammography Screening Service in 2000-2004 with no evidence of a breast lesion. |
| SEARCH | Study of Epidemiology and Risk factors in Cancer Heredity | UK | Identified through the East Anglian Cancer Registry: (i) 1991-1996: alive, prevalent cases diagnosed before age 55; (ii) since 1996: incident cases diagnosed before age 70 diagnosed after 1996. | (a) Women from the same geographic region selected from the EPIC-Norfolk cohort study, 1992-1994 (b) women attending GP practices, frequency matched to cases by age and geographic region (2003-present). |
| UKBGS | UK Breakthrough Generations Study | UK | Cohort members who developed breast cancer or in situ breast cancer after entry into the Breakthrough Generations Study (cohort of >100,000 women followed up for breast cancer, recruited from the UK during 2003-2010). | Women who had not had breast cancer or in situ breast cancer selected by 1:1 matching to cases on date of birth, year of entry in to the study (2003-2010),  source of recruitment, availability of blood sample and ethnicity. |
|  |  |  |  |  |

**Reference List**

1. Dite GS, Jenkins MA, Southey MC, Hocking JS, Giles GG, McCredie MR, Venter DJ, Hopper JL: **Familial risks, early-onset breast cancer, and BRCA1 and BRCA2 germline mutations**. *Journal of the National Cancer Institute* 2003, **95**(6):448-457.

2. Schmidt MK, Tollenaar RA, de Kemp SR, Broeks A, Cornelisse CJ, Smit VT, Peterse JL, van Leeuwen FE, Van't Veer LJ: **Breast cancer survival and tumor characteristics in premenopausal women carrying the CHEK2*1100delC germline mutation**. *Journal of clinical oncology : official journal of the American Society of Clinical Oncology* 2007, **25**(1):64-69.

3. Schrauder M, Frank S, Strissel PL, Lux MP, Bani MR, Rauh C, Sieber CC, Heusinger K, Hartmann A, Schulz-Wendtland R *et al*: **Single nucleotide polymorphism D1853N of the ATM gene may alter the risk for breast cancer**. *Journal of cancer research and clinical oncology* 2008, **134**(8):873-882.

4. Jiang X, Castelao JE, Chavez-Uribe E, Fernandez Rodriguez B, Celeiro Munoz C, Redondo CM, Pena Fernandez M, Novo Dominguez A, Pereira CD, Martinez ME *et al*: **Family history and breast cancer hormone receptor status in a Spanish cohort**. *PloS one* 2012, **7**(1):e29459.

5. Villeneuve S, Fevotte J, Anger A, Truong T, Lamkarkach F, Gaye O, Kerbrat P, Arveux P, Miglianico L, Imbernon E *et al*: **Breast cancer risk by occupation and industry: analysis of the CECILE study, a population-based case-control study in France**. *American journal of industrial medicine* 2011, **54**(7):499-509.

6. Weischer M, Bojesen SE, Tybjaerg-Hansen A, Axelsson CK, Nordestgaard BG: **Increased risk of breast cancer associated with CHEK2*1100delC**. *Journal of clinical oncology : official journal of the American Society of Clinical Oncology* 2007, **25**(1):57-63.

7. Calle EE, Rodriguez C, Jacobs EJ, Almon ML, Chao A, McCullough ML, Feigelson HS, Thun MJ: **The American Cancer Society Cancer Prevention Study II Nutrition Cohort: rationale, study design, and baseline characteristics**. *Cancer* 2002, **94**(9):2490-2501.

8. Widschwendter M, Apostolidou S, Raum E, Rothenbacher D, Fiegl H, Menon U, Stegmaier C, Jacobs IJ, Brenner H: **Epigenotyping in peripheral blood cell DNA and breast cancer risk: a proof of principle study**. *PloS one* 2008, **3**(7):e2656.

9. Justenhoven C, Pierl CB, Haas S, Fischer HP, Baisch C, Hamann U, Harth V, Pesch B, Bruning T, Vollmert C *et al*: **The CYP1B1_1358_GG genotype is associated with estrogen receptor-negative breast cancer**. *Breast cancer research and treatment* 2008, **111**(1):171-177.

10. Pesch B, Ko Y, Brauch H, Hamann U, Harth V, Rabstein S, Pierl C, Fischer HP, Baisch C, Justenhoven C *et al*: **Factors modifying the association between hormone-replacement therapy and breast cancer risk**. *European journal of epidemiology* 2005, **20**(8):699-711.

11. Hartikainen JM, Tuhkanen H, Kataja V, Dunning AM, Antoniou A, Smith P, Arffman A, Pirskanen M, Easton DF, Eskelinen M *et al*: **An autosome-wide scan for linkage disequilibrium-based association in sporadic breast cancer cases in eastern Finland: three candidate regions found**. *Cancer epidemiology, biomarkers & prevention : a publication of the American Association for Cancer Research, cosponsored by the American Society of Preventive Oncology* 2005, **14**(1):75-80.

12. De Maeyer L, Van Limbergen E, De Nys K, Moerman P, Pochet N, Hendrickx W, Wildiers H, Paridaens R, Smeets A, Christiaens MR *et al*: **Does estrogen receptor negative/progesterone receptor positive breast carcinoma exist?** *Journal of clinical oncology : official journal of the American Society of Clinical Oncology* 2008, **26**(2):335-336; author reply 336-338.

13. Flesch-Janys D, Slanger T, Mutschelknauss E, Kropp S, Obi N, Vettorazzi E, Braendle W, Bastert G, Hentschel S, Berger J *et al*: **Risk of different histological types of postmenopausal breast cancer by type and regimen of menopausal hormone therapy**. *International journal of cancer Journal international du cancer* 2008, **123**(4):933-941.

14. Olson JE, Ma CX, Pelleymounter LL, Schaid DJ, Pankratz VS, Vierkant RA, Fredericksen ZS, Ingle JN, Wu Y, Couch F *et al*: **A comprehensive examination of CYP19 variation and breast density**. *Cancer epidemiology, biomarkers & prevention : a publication of the American Association for Cancer Research, cosponsored by the American Society of Preventive Oncology* 2007, **16**(3):623-625.

15. Giles GG, English DR: **The Melbourne Collaborative Cohort Study**. *IARC scientific publications* 2002, **156**:69-70.

16. Garcia-Closas M, Brinton LA, Lissowska J, Chatterjee N, Peplonska B, Anderson WF, Szeszenia-Dabrowska N, Bardin-Mikolajczak A, Zatonski W, Blair A *et al*: **Established breast cancer risk factors by clinically important tumour characteristics**. *British journal of cancer* 2006, **95**(1):123-129.

17. Wedren S, Lovmar L, Humphreys K, Magnusson C, Melhus H, Syvanen AC, Kindmark A, Landegren U, Fermer ML, Stiger F *et al*: **Oestrogen receptor alpha gene haplotype and postmenopausal breast cancer risk: a case control study**. *Breast cancer research : BCR* 2004, **6**(4):R437-449.

18. MacPherson G, Healey CS, Teare MD, Balasubramanian SP, Reed MW, Pharoah PD, Ponder BA, Meuth M, Bhattacharyya NP, Cox A: **Association of a common variant of the CASP8 gene with reduced risk of breast cancer**. *Journal of the National Cancer Institute* 2004, **96**(24):1866-1869.

19. Lesueur F, Pharoah PD, Laing S, Ahmed S, Jordan C, Smith PL, Luben R, Wareham NJ, Easton DF, Dunning AM *et al*: **Allelic association of the human homologue of the mouse modifier Ptprj with breast cancer**. *Human molecular genetics* 2005, **14**(16):2349-2356.

20. Swerdlow AJ, Jones ME, Schoemaker MJ, Hemming J, Thomas D, Williamson J, Ashworth A: **The Breakthrough Generations Study: design of a long-term UK cohort study to investigate breast cancer aetiology**. *British journal of cancer* 2011, **105**(7):911-917.
