# Supplementary material for: Gene–environment interactions involving functional variants: Results from the Breast Cancer Association Consortium
Source: Int J Cancer. 2017 Aug 11;141(9):1830–40. doi: 10.1002/ijc.30859 (PMC5601244; doi:10.1002/ijc.30859)
Supplement: Supplementary file 3 — Supporting Information Table 2. [file IJC-141-1830-s003.docx]

**Supplementary Table 2**. SNP information

| **SNP** | **Minor allele** | **Major allele** | **chromosome** | **region** | **reference** |
| --- | --- | --- | --- | --- | --- |
| rs146784183 | A | G | 1 | 1p11 | [1] |
| rs11249433 | G | A | 1 | 1p11 | [1] |
| rs12405132 | T | C | 1 | RNF115 | [2] |
| rs12048493 | C | A | 1 | OTUD7B | [2] |
| rs72755295 | G | A | 1 | EXO1 | [2] |
| rs7558475 | G | A | 2 | CASP8 | [3] |
| rs36043647 | G | A | 2 | CASP8 | [3] |
| rs1830298 | C | T | 2 | CASP8 | [3] |
| rs59278883 | A | T | 2 | CASP8 | [3] |
| rs6721996 | G | A | 2 | 2q35 | [4] |
| rs4442975 | C | A | 2 | 2q35 | [4] |
| rs6796502 | A | G | 3 | 3p21 | [2] |
| rs73838678 | C | T | 4 | 4q24 | [5] |
| rs62331150 | T | G | 4 | TET2 | [5] |
| rs9790517 | T | C | 4 | TET2 | [5] |
| rs77928427 | A | C | 4 | PPA2 | [5] |
| rs10069690 | A | G | 5 | TERT | [6] |
| rs2242652 | A | G | 5 | TERT | [6] |
| rs7734992 | C | T | 5 | TERT | [6] |
| rs7705526 | A | C | 5 | TERT | [6] |
| rs3215401 | AG | A | 5 | TERT | [6] |
| rs2736108 | A | G | 5 | TERT | [6] |
| rs13162653 | T | G | 5 | 5p15 | [2] |
| rs2012709 | T | C | 5 | 5p13 | [2] |
| rs17432750 | A | C | 5 | MAP3K1 | [7] |
| rs11949391 | C | T | 5 | 5q11.2 | [7] |
| rs62355899 | G | A | 5 | 5q11.2 | [7] |
| rs62355900 | T | C | 5 | 5q11.2 | [7] |
| rs74345699 | T | C | 5 | 5q11.2 | [7] |
| rs62355902 | T | A | 5 | MAP3K1 | [7] |
| rs113317823 | T | C | 5 | MAP3K1 | [7] |
| rs16886397 | G | A | 5 | MAP3K1 | [7] |
| rs7707921 | T | A | 5 | 5q14 | [2] |
| rs150750171 | C | G | 6 | 6p22.1 | [2] |
| rs3757322 | G | T | 6 | CCDC170 | [8] |
| rs2046210 | A | G | 6 | LOC105378058 | [8] |
| rs9397437 | A | G | 6 | LOC105378058 | [8] |
| rs12173570 | T | C | 6 | LOC105378058 | [8] |
| rs851985 | C | A | 6 | ESR1 | [8] |
| rs851984 | A | G | 6 | ESR1 | [8] |
| rs9918437 | T | G | 6 | ESR1 | [8] |
| rs2747652 | T | C | 6 | 6q25 | [8] |
| rs6964587 | T | G | 7 | AKAP9 | [9] |
| rs4593472 | T | C | 7 | 7q32 | [2] |
| rs13365225 | G | A | 8 | 8p11 | [2] |
| rs13267382 | A | G | 8 | LINC00536 | [2] |
| rs35961416 | CA | C | 8 | 8q24 | [10] |
| rs7815245 | T | C | 8 | CASC21 | [10] |
| rs2033101 | T | C | 8 | PVT1 | [10] |
| rs1121948 | G | A | 8 | LOC105375755 | [10] |
| rs11780156 | T | C | 8 | LOC105375755 | [10] |
| rs10816625 | G | A | 9 | 9q31.2 | [11] |
| rs13294895 | A | G | 9 | 9q31.2 | [11] |
| rs676256 | G | A | 9 | 9q31.2 | [11] |
| rs145759243 | TGAA | T | 10 | ZNF365 | [12] |
| rs10995194 | C | G | 10 | ZNF365 | [12] |
| rs7922449 | T | C | 10 | ZNF365 | [12] |
| rs10995201 | G | A | 10 | ZNF365 | [12] |
| rs9971363 | G | A | 10 | ZNF365 | [12] |
| rs2981578 | C | T | 10 | FGFR2 | [13] |
| rs35054928 | GC | G | 10 | FGFR2 | [13] |
| rs45631563 | T | A | 10 | FGFR2 | [13] |
| rs78540526 | T | C | 11 | 11q13 | [14] |
| rs554219 | G | C | 11 | 11q13 | [14] |
| rs494406 | A | G | 11 | 11q13 | [14] |
| rs75915166 | A | C | 11 | 11q13 | [14] |
| rs11627032 | C | T | 14 | 14q32 | [2] |
| rs146699004 | G | GGT | 17 | TEFM | [2] |
| rs745570 | G | A | 17 | 17q25.3 | [2] |
| rs6507583 | G | A | 18 | SETBP1 | [2] |

1. Horne, H.N., et al., *Fine-Mapping of the 1p11.2 Breast Cancer Susceptibility Locus.* PLoS One, 2016. **11**(8): p. e0160316.

2. Michailidou, K., et al., *Genome-wide association analysis of more than 120,000 individuals identifies 15 new susceptibility loci for breast cancer.* Nat Genet, 2015. **47**(4): p. 373-80.

3. Lin, W.Y., et al., *Identification and characterization of novel associations in the CASP8/ALS2CR12 region on chromosome 2 with breast cancer risk.* Hum Mol Genet, 2015. **24**(1): p. 285-98.

4. Ghoussaini, M., et al., *Evidence that breast cancer risk at the 2q35 locus is mediated through IGFBP5 regulation.* Nat Commun, 2014. **4**: p. 4999.

5. Guo, X., et al., *Fine-Scale Mapping of the 4q24 Locus Identifies Two Independent Loci Associated with Breast Cancer Risk.* Cancer Epidemiol Biomarkers Prev, 2015. **24**(11): p. 1680-91.

6. Bojesen, S.E., et al., *Multiple independent variants at the TERT locus are associated with telomere length and risks of breast and ovarian cancer.* Nat Genet, 2013. **45**(4): p. 371-84, 384e1-2.

7. Glubb, D.M., et al., *Fine-scale mapping of the 5q11.2 breast cancer locus reveals at least three independent risk variants regulating MAP3K1.* Am J Hum Genet, 2015. **96**(1): p. 5-20.

8. Dunning, A.M., et al., *Breast cancer risk variants at 6q25 display different phenotype associations and regulate ESR1, RMND1 and CCDC170.* Nat Genet, 2016. **48**(4): p. 374-86.

9. Milne, R.L., et al., *Common non-synonymous SNPs associated with breast cancer susceptibility: findings from the Breast Cancer Association Consortium.* Hum Mol Genet, 2014. **23**(22): p. 6096-111.

10. Shi, J., et al., *Fine-scale mapping of 8q24 locus identifies multiple independent risk variants for breast cancer.* Int J Cancer, 2016.

11. Orr, N., et al., *Fine-mapping identifies two additional breast cancer susceptibility loci at 9q31.2.* Hum Mol Genet, 2015. **24**(10): p. 2966-84.

12. Darabi, H., et al., *Polymorphisms in a Putative Enhancer at the 10q21.2 Breast Cancer Risk Locus Regulate NRBF2 Expression.* Am J Hum Genet, 2015. **97**(1): p. 22-34.

13. Meyer, K.B., et al., *Fine-scale mapping of the FGFR2 breast cancer risk locus: putative functional variants differentially bind FOXA1 and E2F1.* Am J Hum Genet, 2013. **93**(6): p. 1046-60.

14. French, J.D., et al., *Functional variants at the 11q13 risk locus for breast cancer regulate cyclin D1 expression through long-range enhancers.* Am J Hum Genet, 2013. **92**(4): p. 489-503.
